# A *Guide *for applying a revised version of the PARIHS framework for implementation

**DOI:** 10.1186/1748-5908-6-99

**Published:** 2011-08-30

**Authors:** Cheryl B Stetler, Laura J Damschroder, Christian D Helfrich, Hildi J Hagedorn

**Affiliations:** 1 Independent Consultant, Amherst, Massachusetts, USA; 2 Health Services Department, Boston University School of Public Health, Boston, Massachusetts, USA; 3 HSR&D Center for Clinical Management Research and Diabetes QUERI, VA Ann Arbor Healthcare System, Ann Arbor, Michigan, USA; 4 Northwest HSR&D Center of Excellence, VA Puget Sound Healthcare System, Seattle, Washington, USA; 5 Department of Health Services, University of Washington School of Public Health, Seattle, Washington, USA; 6 VA Substance Use Disorders Quality Enhancement Research Initiative, Minneapolis VA Medical Center, Minneapolis, Minnesota, USA; 7 Department of Psychiatry, School of Medicine, University of Minnesota, Minneapolis, Minnesota, USA

## Abstract

**Background:**

Based on a critical synthesis of literature on use of the Promoting Action on Research Implementation in Health Services (PARIHS) framework, revisions and a companion *Guide *were developed by a group of researchers independent of the original PARIHS team. The purpose of the *Guide *is to enhance and optimize efforts of researchers using PARIHS in implementation trials and evaluations.

**Methods:**

Authors used a planned, structured process to organize and synthesize critiques, discussions, and potential recommendations for refinements of the PARIHS framework arising from a systematic review. Using a templated form, each author independently recorded key components for each reviewed paper; that is, study definitions, perceived strengths/limitations of PARIHS, other observations regarding key issues and recommendations regarding needed refinements. After reaching consensus on these key components, the authors summarized the information and developed the *Guide*.

**Results:**

A number of revisions, perceived as consistent with the PARIHS framework's general nature and intent, are proposed. The related *Guide *is composed of a set of reference tools, provided in Additional files. Its core content is built upon the basic elements of PARIHS and current implementation science.

**Conclusions:**

We invite researchers using PARIHS for targeted evidence-based practice (EBP) implementations with a strong task-orientation to use this *Guide *as a companion and to apply the revised framework prospectively and comprehensively. Researchers also are encouraged to evaluate its use relative to perceived strengths and issues. Such evaluations and critical reflections regarding PARIHS and our *Guide *could thereby promote the framework's continued evolution.

## Background

In October 2010, a critical synthesis of literature on the use of the Promoting Action on Research Implementation in Health Services (PARIHS) framework was published in *Implementation Science *[1]. PARIHS is a widely cited conceptual framework that conceives of three key, interacting elements that influence successful implementation of evidence-based practices (EBPs): *Evidence *(*E*), *Context *(*C*), and *Facilitation *(*F*). The literature synthesis identified key strengths and issues as regards the framework.

A subgroup of the synthesis authors drew upon the above results to revise PARIHS for use by researchers in the Veteran's Health Administration (VA); that is, in trials or evaluations focused on implementation of targeted EBPs. A companion document, or *Guide*, also was developed to provide direction on how this revised version could be operationalized. Together, the framework modifications and *Guide *addressed barriers to the use of PARIHS previously encountered by VA researchers, in part due to the framework's limitations [1]. It is important to note that although we propose a number of revisions and comment on how best to use PARIHS, we have built on the original work of the PARIHS team [2-5]; and while we have shared our work with members of that team, this version of PARIHS and our related *Guide *were developed independently. It does not necessarily reflect the PARIHS team's views. This work further reflects our efforts to operationalize the PARIHS framework based on our VA research context, our VA experience with PARIHS, and our critical review [1]. Were others to follow the same process, they might come to different interpretations and conclusions.

Our *Guide *is intended to enhance and optimize the efforts of those choosing to use PARIHS as their theoretical framework. It is designed to enable users to more clearly and consistently define and apply relevant terms. Further, it is designed to facilitate diagnostic analysis of framework elements, selection of an appropriate implementation strategy, and measurement of *Successful Implementation*. It is hoped that similar syntheses and guides will be developed for other implementation theories, models, and frameworks [6]. Within the VA, where no single theory takes precedence over any other, efforts are underway to enhance operationalization of other frameworks and models by mapping their elements to constructs identified through a Consolidated Framework for Implementation Research (CFIR)[7].

Since the intent of this paper is to provide others interested in using PARIHS with tool-based, practical guidance, we rely heavily on additional files. These files equip users of the framework with the following: a set of definitions for elements/sub-elements, tips in the form of observations about use of elements/sub-elements, and a set of questions for diagnostic analysis and planning. All of the separate components of the actual *Guide *are contained in additional files (see Additional Files 1, 2, 3 and 4). The main narrative provides only overview information and pointers regarding various *Guide *components. Specifically, this overview briefly describes the basic underlying PARIHS framework [2-5], its limitations and related issues [1], the structured process and frames of reference used to identify modifications and create the *Guide*, and the revisions to the original framework [2-5]. It also provides sample material from additional files to give readers a better feel for their content and potential usefulness.

### Brief overview of PARIHS

PARIHS can be characterized as an *impact or explanatory *framework [6], originally developed in 1998 [8] and refined over time based on concept analyses and exploratory research [2-5,9,10].

**Figure 1:**
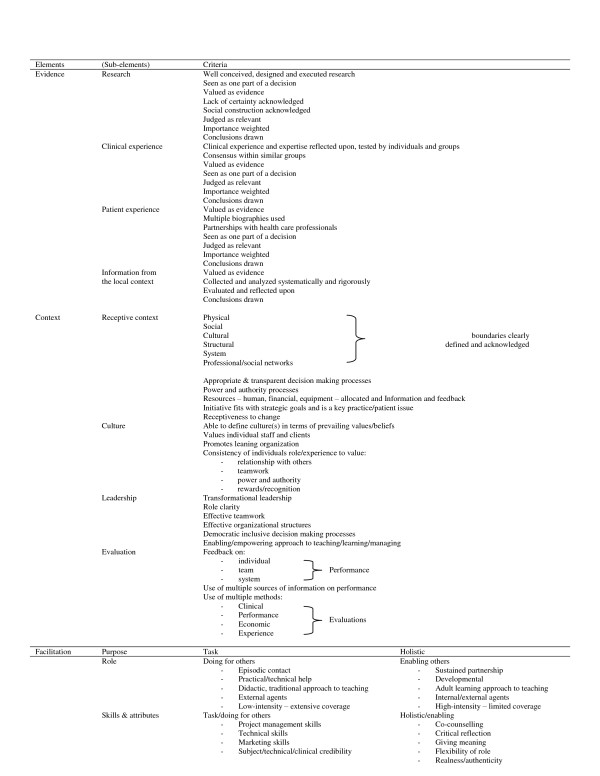
**Key elements for implementing evidence into practice **[3]. This figure reproduces the PARIHS team's 2004 version of its framework, with all its elements and sub-elements and "criteria," from the following publication: Rycroft-Malone J, Harvey G, Seers K, Kitson A, McCormack B, Titchen A: An exploration of the factors that influence the implementation of evidence into practice. *J Clin Nurs*, 2004, **13**(8): 913-924. It is reproduced with permission. "Criteria" highlight the conditions more likely needed for, or critical to, successful implementation.

Before using our *Guide*, it is important that users be familiar with the underlying framework of PARIHS [2,3,5] (*e.g*., see Rycroft-Malone *et al*. [3] for a recent depiction of the framework, including its key sub-elements and explanatory material; also see Kitson *et al*.'s discussion regarding theoretical issues in general and PARIHS' status specifically, noting the potential diagnostic and evaluative questions they provide in a related appendix [5]). Another, more recent publication provides an overview of the framework, its underlying assumptions, developmental work, and its use by others [11]. Key aspects of the PARIHS framework are herein summarized in Table 1. Figure 1 outlines the sub-elements of each of the core elements, as described in the PARIHS team's 2004 refinement [3].

**Table 1:** Description of the underlying PARIHS framework [2-5]

Purpose	"...to provide a map to enable others to make sense of [the] complexity [of implementation], and the elements that require attention if implementation is more likely to be successful" [5]
Proposition	*Successful Implementation (SI) is a (f)unction of Evidence (E), Context (C), and Facilitation (F)*. The actual complexity of this formula is represented in the framework through the following:
	• Its numerous, potentially applicable sub-elements within its three overarching elements
	• Its recognition of the nature of complex and dynamic inter-relationships among *E, C*, and *F *

Core elements	• *Evidence *(*E*) = "codified and non-codified sources of knowledge," as perceived by multiple stakeholders
	• *Context *(*C*) = quality of the environment or setting in which the research is implemented
	• *Facilitation *(*F*) = a "technique by which one person makes things easier for others," achieved through "support to help people change their attitudes, habits, skills, ways of thinking, and working"
	Each element can be assessed for whether its status is weak ("low" rating) or strong ("high" rating) and thus can have a negative or positive influence on implementation. For *Facilitation*, the focus is on rating "appropriateness."

PARIHS = Promoting Action on Research Implementation in Health Services.

In summary, PARIHS can be selected as a broad framework to guide development of a program of implementation interventions that effectively enable EBP-related changes. Specifically, it can be used to diagnose critical elements related to implementation of an EBP (*E *and *C*) and thence development of an implementation strategy (*F*) to enable successful and sustained change. A PARIHS-based diagnostic analysis can additionally engage stakeholders in self-reflection regarding critical aspects of implementation and the related nature of needed change [12].

### PARIHS limitations and related issues

Strengths of the PARIHS framework identified through our published synthesis included the following: its intuitive appeal, provision of a basic "to-do" list, flexibility in application, and inclusion of *Successful Implementation *as the desired outcome [1]. Of particular importance to development of the *Guide *were its identified limitations and related issues [1]. These included the following, which are further described in Table 2:

**Table 2:** Limitations of and related issues with the underlying PARIHS framework [1]

Conceptual clarity	• Ambiguity in certain terms and phrases; for example, when assessing *Evidence*, one criterion for "high" research evidence is that "social construction [is] acknowledged." Cross-country and philosophical differences may contribute to this perception of "obscurity" in such language.
	• Lack of specificity in element/sub-element names and definitions, making it unclear what is actually included/excluded; for example, one of the elements is titled *Context*, as is one of its sub-elements, *Receptive Context*.
	• Lack of transparency or specificity in how to operationalize various sub-elements, such as clinical experience or patient experience.
"Missing" components	• Lack of a definition for *Successful Implementation *(SI).
	• Need to explicitly designate *motivation for change*/importance of a "recognized need for change" [34], as pointed out by Ellis *et al*.
	• Potential value of making more explicit a critical set of innovation attributes (*e.g*., per Rogers' diffusion of innovation theory [33]).
	• Removal of clearly stated attributes of a facilitator after earliest version of PARIHS (*i.e*., general credibility, authenticity, and respect).
	• Insufficient guidance or clarification under *Facilitation *regarding the task of developing needed "change...strategies" [5], based on suggested diagnostic analysis of *E *and *C*--and lack of inclusion of common implementation interventions that a Facilitator employs, reinforces, or proposes to enhance adoption.

Under-developed evaluation and related instrumentation/measures	• Few well-developed PARIHS-related instruments or other evaluative approaches to identify related barriers/facilitators during diagnostic analysis or to evaluate successful implementation.
	• Limited evaluation or means for evaluation of the theory's use/usefulness.

PARIHS = Promoting Action on Research Implementation in Health Services.

• Lack of conceptual clarity, specificity, and transparency, which results in different interpretations of PARIHS concepts by different researchers

• Lack of inclusion of relevant elements perceived to be critical to implementation and congruent with the main intent of PARIHS

• Lack of well-developed instrumentation and evaluation measures, as well as limited evaluation of actual use or perceived usefulness of the framework.

No published studies were identified that used the framework comprehensively and prospectively to develop an implementation project. The ability to fully evaluate its usefulness thus has been limited.

## Methods

### Revising PARIHS for use in task-oriented implementation

Our objective in developing the *Guide *was to meet the needs of VA researchers interested in understanding the nuts and bolts of operationalizing PARIHS. More specifically, our objective was two-fold: (1) provide guidance on *how best to apply/operationalize the framework within QUERI's *[Quality Enhancement Research Initiative] *action-oriented approach *[13-15] and (2) enable more effective use of the framework by addressing identified barriers (Table 2). (Note: *Italicized *sentences here and in the next section come from our internal PARIHS synthesis/application project plan.)

Given this practical need, after completion of the synthesis groundwork, the authors used a planned, structured process to *organize and bring together into a coherent whole the substance of our critiques, related discussions, and potential recommendations for refinements/adaptations *of the PARIHS framework--*for use within the context of QUERI-like implementation projects*. Specifically, the authors did the following:

1) Utilizing finalized critiques from the published synthesis [1], each author independently recorded key components for each reviewed paper on a templated form. This form focused on the study's definition of elements, perceived strengths/limitations of PARIHS highlighted by the study, other observations regarding key PARIHS issues, and recommendations regarding *refinements consistent with the intent of the basic framework ... in light of the QUERI framework, QUERI experience and current science*.

2) Each author independently reviewed selected components of two other published syntheses that analyzed the concept of *Context *[7,16].

3) As a group, the authors critically reviewed, discussed, and themed the above information at a two-day intensive face-to-face meeting.)

4) As a group, the authors reached consensus on the above key components, including the clarity/lack of clarity of language found in various definitions, and then identified opportunities to improve the framework.

Information from step 4 was used to draft a *Guide*. Critical to this draft was the original PARIHS framework, primarily its two most recent versions [2,3] and the 2008 paper and Appendix [5]. Feedback was obtained from VA implementation researchers [1] and others familiar with PARIHS, and minor refinements made.

Critical to understanding the general implementation approach embedded within this *Guide *is the nature of QUERI's action-oriented paradigm. This implementation/research paradigm served as an implicit background or frame of reference for overall author deliberations. It distinguishes two general types of implementation situations and emphasizes a set of innovative concepts.

#### Types of implementation

We distinguish two general types of implementation situations:

• one with a *task-oriented *purpose, where a specific intervention is being implemented within a relatively short timeframe (such as implementing a new procedure or care process)

• one with a broader *"organizational" *purpose, where implementation strategies are targeted at transformational change within one or more levels of an institution (such as changing culture to be more receptive to using EBPs on a routine basis [17]).

The primary focus of QUERI projects, and thus the purpose of this *Guide*, is to assist with more short-term, targeted EBP implementation studies with a strong task orientation [14,15]. We highlight this distinction because it influenced how we approached framework refinements and identified observations/tips in the reference tools.

In short-term, task-oriented situations, implementation efforts are unlikely to target broad changes in the multiple sub-elements related to culture, evaluation, or leadership. We therefore focused on defining and highlighting only those aspects of PARIHS elements that might realistically be modified in a relatively short period of time.

It is important to further distinguish our use of the terms *task *versus *organizational *purpose from the PARIHS framework's approach to *Facilitation*. The latter envisages the *purpose *of *Facilitation *to occur along a continuum from primarily "task" to "holistic." The former focuses on "a 'doing for others' role ... [and is] more discrete, practical, technical and task driven," while the latter focuses on "an 'enabling and empowering' role which is more developmental" [5]. In most cases, task-oriented EBP implementation situations will rely more heavily on task-focused or "mixed" *Facilitation *methods; on the other hand, transformational initiatives that have an organizational redesign goal will rely more heavily on holistic *Facilitation *[5].

#### Innovative, action-oriented QUERI concepts

As QUERI developed over time, a set of concepts guided its implementation research activities. Some of these concepts relate to QUERI innovations or contributions [7,14,15,17-24], others to the Stetler model of EBP [25,26], and yet others to the general implementation science literature spanning the last decade [16,27-33]. Such concepts include, for example, strength of evidence, theoretical underpinnings, attributes of innovations, appropriate variation and qualifiers for use of evidence, social marketing and other recognized implementation interventions, sustainability, cost considerations for implementation, and critical leadership behaviors. Such concepts were familiar to the authors, were implicitly part of our decision-making, and ultimately influenced our development of the *Guide's *content in general and construction of the files' "Related Observations/Tips" most specifically.

## Results

### Revisions to PARIHS

Based on the above process and frames of reference, a number of modifications were made to the original PARIHS framework. Emphasis was placed on modifiable sub-elements or ones that might be buffered to reduce negative influences. This revised version of PARIHS is outlined in Table 3. Of particular note are the following:

**Table 3:** Revised PARIHS framework for a task-oriented approach to implementation: *SI *= function of *E, C, F*

Elements	Sub-elements
*E*: *Evidence and EBP Characteristics*	• Research and published guidelines
	• Clinical experiences and perceptions
	• Patient experiences, needs, and preferences
	• Local practice information
	• Characteristics of the targeted EBP:
	• Relative advantage
	• Observability
	• Compatibility
	• Complexity
	• Trialability
	• Design quality and packaging
	• Costs

*C*: *Contextual Readiness for Targeted EBP Implementation*	• Leadership support
	• Culture
	• Evaluation capabilities
	• Receptivity to the targeted innovation/change

*F*: *Facilitation*	*Role of facilitator:*
	• Purpose, external and/or internal role
	• Expectations and activities
	• Skills and attributes of facilitator
	*Other implementation interventions *suggested per site diagnostic assessment or relevant sources (*e.g*., prior research/literature and supplementary theories) and used by the *Facilitator *and others
	• Related to *E*
	• Related to *C*
	• Other

*SI*: *Successful Implementation*	• Implementation plan and its realization
	• EBP innovation uptake: uptake of clinical interventions and/or delivery system interventions
	• Patient and organizational outcomes achievement

PARIHS = Promoting Action on Research Implementation in Health Services; EBP = evidence-based practice.

• Changes were made both to wording and ordering of a few elements/sub-elements, as can be seen in comparing Table 3 to Figure 1. For example, the name of the *Context *element was amended (*Contextual Readiness for Targeted EBP Implementation*) to clearly indicate our task-oriented focus; and *Leadership *became the first sub-element under *Context*, indicating its prime importance in implementation. Nonetheless, it is important to note that the original PARIHS sub-elements of transformational leadership are still reflected within the *Guide *(*e.g*., role clarity and effective teamwork).

• A few items were added to core elements to reflect relevant features critical to implementation but missing from the framework (Table 2); for example, *EBP Characteristics *within *Evidence *now highlights attributes of an implementable form of "evidence" (*i.e*., the full form of an "EBP" innovation, such as a policy, procedure, or program). These additions were drawn from Roger's diffusion of innovation work [33] and the CFIR [7]. Some of these additions were already implicit within other *Evidence *sub-elements. As a result there may appear to be some overlap. However, these attributes were considered important enough to be expanded and made explicit, thus ensuring their consideration. This is particularly important because implementation decisions flow first from the nature of the implementable form of the *Evidence *and its characteristics.

Additionally, for *Facilitation*, implementation interventions beyond that of a facilitator role were inserted. This modification speaks in part to the 2008 PARIHS paper's comment regarding development of a "programme of change," that is, "task based, planned change programme approaches that meet the individual and team's learning needs...." [5]--and, we would add, that meet contextual needs identified through diagnostic analysis. As these programmes of change are likely to require "a range of different techniques" [5], we now make such techniques more explicit. This ties "*Facilitation *as an intervention" [5] to implementation interventions in general, which facilitators and others employ to enhance adoption.

• *Successful Implementation *is now visualized as an explicit part of the revised PARIHS "figure" (Table 3), with detailed definitions provided in the *Guide *(Additional File 4). This first effort at explicating the meaning of *Successful Implementation *is only preliminary and will benefit from ongoing attempts to operationalize it.

Finally, based on our synthesis, our frames of reference, and our framework modifications, we were able to construct a *Guide *(Table 4). Again, its intent is to enhance and optimize efforts of those using PARIHS as their theoretical framework. Within the *Guide*, the team used active, pragmatic language for each element/sub-element--and, again, tied these changes to the original PARIHS framework material and its perceived intent. Such language focuses on recognizable, measurable behaviors and minimizes what to us was abstract language less familiar to our researchers. The content of all additional files provides the following:

**Table 4:** Additional files: *Guide *for applying a revised version of the PARIHS framework for implementation

A. Additional File 1: "EVIDENCE" Element: *Evidence and EBP Characteristics *(*E*)
• *E *element and related sub-elements
• Conceptual definitions
• Detailed observations/tips regarding sub-elements and measurement
• Sample, optional questions to guide formative evaluation

B. Additional File 2: "CONTEXT" Element: *Contextual Readiness for Targeted EBP Implementation *(*C*)
• *C *element and related sub-elements
• Conceptual definitions
• Detailed observations/tips regarding sub-elements and measurement
• Sample, optional questions to guide formative evaluation

C. Additional File 3: "FACILITATION" (*F*) Element
• *F *element and related sub-elements
• Conceptual definitions
• Detailed observations/tips regarding sub-elements and measurement
• Sample, optional questions to guide the team's project planning

D. Additional File 4: "SUCCESSFUL IMPLEMENTATION" (*SI*) Element
• *SI *sub-elements
• Conceptual definitions
• Detailed observations/tips regarding sub-elements and measurement
• Sample, optional questions to guide the team's development of an evaluation plan

PARIHS = Promoting Action on Research Implementation in Health Services; EBP = evidence-based practice.

• Conceptual and operational definitions: This includes refined meanings of constructs within the framework, reflecting the team's interpretation of each element and related sub-element. These definitions are intended to facilitate in-depth understanding of each concept, guide application of the various elements, and identify potential questions for diagnostic analysis and planning.

• Observations and tips: This additional information, from the implementation literature and authors' experiences, is designed to enhance researchers' nuanced understanding of PARIHS elements/sub-elements. Tips also may facilitate design decisions.

As stated previously, the material contained across the additional files (*i.e*., the revised PARIHS *Guide*) is the meat of this publication. It is intended to be used as an active reference tool for planning implementation research and evaluation. Tables 5, 6 and 7 provide the reader with a preview of these reference tools. Table 5 points out how we describe the potential use of an individual tool; Table 6 illustrates our approach to defining each of the core elements; and Table 7 demonstrates how an individual sub-element is presented in terms of its definitions, tips on use, and measurement.

**Table 5:** Illustration of *Guide *content: description of potential uses of a sample tool

Element	Reference tool content
*C*: *Contextual Readiness for Targeted EBP Implementation*	Information in this and the other tools in this *Revised PARIHS Guide *can be used to prepare a proposal, including related methodology, and follow-up reports. More specifically, this *Context *tool can be used to:
• Leadership support	• Think more specifically about the nature of *Context *and enhance communication of that understanding to reviewers and other readers.
• Culture	• Identify potential *Contextual *barriers that may need to be better understood and/or addressed in the implementation strategy (*e.g*., thinking through the type of leadership support that will be needed given the type of innovation to be implemented).
• Evaluation capabilities	• Identify diagnostic/evaluative questions for a semi-structured interview relevant to the need to understand selected aspects of the *Context*, applicable to this specific EBP change.
• Receptivity to the targeted innovation/change	• Develop and organize a retrospective interpretive evaluation [20] to explore the perceived influence of *Contextual *features on implementation of the targeted EBP.
	*NOTE: In all cases, the list of multiple items should be considered an optional menu from which to choose components of prime relevance to implementation of the targeted EBP*.

PARIHS = Promoting Action on Research Implementation in Health Services; EBP = evidence-based practice.

**Table 6:** Illustration of *Guide *content: description of a core element

Element	Conceptual definitions	Related observations/tips	Measurement
*Evidence & EBP Characteristics*	***Evidence *****= **Specified sources of information relevant to a specific EBP, including research/published guidelines, clinical experience, patient experience, and/or local practice information.	As "evidence" is socially constructed [4], the perceptions of targeted stakeholders regarding the nature and quality of these varying sources of evidence are key to development of an implementation strategy.	Two quantitative measurement instruments have been developed that incorporate major components of PARIHS related to *Evidence*: ORCA [18] and a survey developed by Bahtsevani and colleagues [35].
	• These sources have presumably been subjected to scrutiny (*e.g.*, by the research team or a national body) and are judged to support or refute effectiveness of a targeted EBP intervention/recommendation.	• This includes perception of the form of the evidence-based clinical recommendation/intervention (*i.e.*, the recommended practice as a guideline, policy, procedure, protocol, program, optional or forced function clinical reminder, decision algorithm, etc.). At times such transformed findings/"evidence" is supplemented with additional content based on the judgment or consensus of its creator (*e.g.*, consider the mixed nature of various guidelines or protocols).	Sample qualitative diagnostic questions for use in task-oriented projects are listed for each element/sub-element and are, for the most part, based on adaptations of items from the Kitson *et al*. Appendix related to *Evidence *[5]. Their 2008 Appendix is said to outline "diagnostic and evaluative measures," but it is not a formal "tool."
	***EBP Characteristics ***= Attributes describing the nature of the implementable form of the evidence/practice recommendation.	• Perceptions of key stakeholders can be influenced by various attributes [7,33] related to this EBP and its evidentiary source/s.	• Initial, diagnostic evaluation is herein referenced as the first stage of an implementation project's formative evaluation [20].

PARIHS = Promoting Action on Research Implementation in Health Services; EBP = evidence-based practice; ORCA = Organizational Readiness to Change Assessment.

**Table 7:** Illustration of *Guide *content: sample material for a sub-element

Related Sub-elements	Conceptual definitions	Detailed observations regarding sub-elements	Sample, optional questions to guide formative evaluation
*Leadership support*	***Leadership = ***Individuals in designated positions "...at any level of the organization including executive leaders, middle management, front-line supervisors, and team leaders, who have a direct or indirect influence on the implementation" [7] ***Leadership Support **= *Behaviors, [*verbalized*] attitudes, and actions of leaders that reflect readiness or receptivity to a change [17]	• In general, relevant leaders' "supportive" *actions *can be characterized by various types of managerial behaviors or responsibilities, within a change/innovation situation such as EBP, as listed below. These are not directly taken from the original PARIHS framework but rather have been adapted based on the following: a task-oriented view of related PARIHS sub-elements, supplemental information from relevant papers [17][36,37], relevant EBP behaviors of transformational leaders [17], and an effort to use language more familiar to targeted researchers. • *Role clarity, e.g.*, ensuring transparency regarding both *project*-related and relevant *change*-related role responsibilities and accountabilities.	• To what extent do leaders show active and visible support for this change or this type of EBP and implementation? ○ Is the leader willing to engage with the study team for planning? ○ Is the leader willing to provide connections/entrees for the study team? ○ Does the leader have experience/comfort in this role? ○ Does the leader hold service directors accountable for collaboration and coordination in such change efforts/in this effort? • To what extent are appropriate stakeholders or teams held accountable and incentivized or rewarded to carry out the implementation? ○ What about past experiences with this *type *of change? • To what extent does the leader indicate the willingness to and in fact does the leader communicate the priority of this implementation?

PARIHS = Promoting Action on Research Implementation in Health Services; EBP = evidence-based practice.

### Summary and conclusions

Based on a systematic, structured process, the authors have revised PARIHS and provided a detailed reference *Guide *to help researchers apply this framework. When using the *Guide*, readers should keep the following points in mind:

• The *Guide *relies on basic elements of PARIHS, as well as updates provided in Kitson and colleagues' 2008 paper and its appendix, specifically its diagnostic approach [5].

• A key revision objective was to minimize the original framework's limitations and related issues (Table 2).

• Our modifications are consistent with the general nature and intent of the PARIHS framework.

• Basic expectations for applying any framework, theory, or model were a guiding influence, that is, the need for clear conceptual and operational definitions, measurement approaches, and additional practical information about the realities of application.

• QUERI frames of reference and concepts affected development of *Guide *content, as did supplemental information from complementary theories such as Rogers, the Stetler model of EBP, and other selected concepts from implementation science. Modifications are thus responsive to the PARIHS team's suggestion [5] to draw on other theoretical perspectives; for example, "What theories would inform the way evidence has been conceptualized within the PARIHS framework?"

• The implementation knowledge and experience-based lessons of the author team (published implementation scientists in the VA) influenced consensual judgments underlying the *Guide*.

• Our addition of "other implementation interventions" to the *Facilitation *element draws, in part, from a QUERI evaluation on facilitation wherein data suggested the following: "external facilitators were likely to use or integrate other implementation interventions, while performing this problem-solving and supportive role" [19].

The *Guide *has been disseminated within the VA as a resource for implementation scientists. Individuals familiar to the authors (personal communications) have reported using the modified framework in their studies or intending to put it to use in the near future. Such uses included the following:

• Guiding new investigators looking for "theoretical" assistance

• Simplifying selection of diagnostic/evaluative questions relevant to a targeted EBP, followed by organization of those questions into a semi-structured interview

• Defining specifics of an external facilitation intervention (*e.g*., the level of interaction and type of external facilitator needed), thus making formative evaluation easier [20]

• Facilitating thinking about what *Successful Implementation *would look like in a study and how that would be measured

• Assisting in the preparation of a proposal wherein use of a theoretical framework and related design decisions could more clearly be explained to reviewers.

In conclusion, the PARIHS synthesis paper suggested that "the single greatest need for researchers using PARIHS, and other implementation models, is to use the framework prospectively and comprehensively, and evaluate that use relative to its perceived strengths and issues for enhancing successful implementation" [1]. Those using this manuscript to either implement a targeted EBP or study such an implementation thus are encouraged to use the *Guide *prospectively/comprehensively and to evaluate its use. Formal evaluations and critical reflections regarding the usefulness and limitations of our revised PARIHS and *Guide *could thereby promote continued evolution of this promising framework.

## Competing interests

The authors declare that they have no competing interests.

## Authors' contributions

CBS conceived the design of a *Guide *and drafted both the initial manuscript and *Guide*. All authors contributed to development of its content, reviewed drafts, and provided major input and revisions. All authors approved the final manuscript.

## Supplementary Material

Additional file 1 **EVIDENCE REFERENCE TOOL: Definitions for a "Revised" *EVIDENCE *Element: *EVIDENCE & EBP CHARACTERISTICS***. This *Evidence *Reference Tool provides explicit definitions for this element and its sub-elements; related, detailed explanations and observations; and sample, optional questions to guide formative evaluation. Click here for file

Additional file 2 **CONTEXT REFERENCE TOOL: Definitions for a "Revised" PARIHS "*Context*" Element: *Contextual Readiness for Targeted EBP Implementation***. This *Context *Reference Tool provides explicit definitions for this element and its sub-elements; related, detailed explanations and observations; and sample, optional questions to guide formative evaluation. Click here for file

Additional file 3 **FACILITATION REFERENCE TOOL: Definitions for a "Revised" PARIHS *FACILITATION *Element**. This Facilitation Reference Tool provides explicit definitions for this element and its sub-elements; related, detailed explanations and observations; and sample, optional questions to guide planning the critical details of implementation. Click here for file

Additional file 4 **SUCCESSFUL IMPLEMENTATION TOOL: Definitions for a "Revised" PARIHS *Successful Implementation *Element**. This Successful Implementation Reference Tool provides information on three foci for evaluation of this component of the framework; related definitions and key issues; and observations and suggestions regarding relevant measurements. Click here for file
