# Supplementary material for: A Guide for applying a revised version of the PARIHS framework for implementation
Source: Implement Sci. 2011 Aug 30;6:99. doi: 10.1186/1748-5908-6-99 (PMC3184083; doi:10.1186/1748-5908-6-99)
Supplement: Additional file 1 — EVIDENCE REFERENCE TOOL: Definitions for a "Revised" EVIDENCE Element: EVIDENCE & EBP CHARACTERISTICS. This Evidence Reference Tool provides explicit definitions for this element and its sub-elements; related, detailed explanations and observations; and sample, optional questions to guide formative evaluation. [file 1748-5908-6-99-S1.PDF]

EVIDENCE REFERENCE TOOL: Definitions for a "Revised" *EVIDENCE* Element: *EVIDENCE & EBP CHARACTERISTICS*

A "Revised PARIHS Framework": Elements & Sub-Elements for a Task-Oriented Approach to Implementation: SI = f (E,C,F)

<table>
<tr>
<td>

E: *Evidence and EBP Characteristics:*

- ***Research and published guidelines***
- ***Clinical experiences and perceptions***
- ***Patient experiences, needs and preferences***
- ***Local practice information***
- ***Characteristics of the targeted EBP***

</td>
<td>

Reference Tool Content

Information in this and the other tools in this *Revised PARIHS Guide* can be used to prepare a proposal, including related methodology, and follow-up reports. More specifically, this *Evidence* tool can be used to:

- Clarify the team's understanding of the *Evidence* element and enhance communication of that understanding to reviewers and other readers
- Identify relevant issues that may need to be addressed or better understood before creating a diagnostic analysis or implementation strategy (e.g., thinking through the form, nature and characteristics of the evidence/recommended EBP, a critical first step)
- Select diagnostic/evaluative questions relevant to the above understanding of the targeted EBP and organize those questions into a semi-structured interview/focus group guide. This tool can thus make it easier to identify potential barriers and facilitators to implementation related to the *Evidence* element, and thereby facilitate construction or refinement of an implementation strategy.
- Develop and organize a retrospective interpretive evaluation [20] to explore the perceived influence of the sources and characteristics of the *Evidence*/EBP on its implementation.

NOTE: In all cases, lists of multiple items should be considered an optional menu from which to choose components of prime relevance to implementation of the targeted EBP.

</td>
</tr>
</table>

C: *Contextual Readiness for Targeted EBP Implementation*

- *Leadership support*
- *Culture*
- *Evaluation capabilities*
- *Receptivity to the targeted innovation/change*

F: *Facilitation*

- *Role of facilitator:*
  - *Purpose, external and/or internal role*
  - *Expectations and activities*
  - *Skills and attributes of facilitator*
- *Other implementation interventions*
  - *Related to "E"*
  - *Related to "C"*
  - *Other*

SI: *Successful Implementation/Metrics and Indicators*

- *Implementation plan and its realization*
- *EBP innovation uptake: i.e., of clinical interventions and/or delivery system interventions*
- *Patient and organizational outcomes achievement*

EVIDENCE REFERENCE TOOL: Definitions for a "Revised" *EVIDENCE* Element: *EVIDENCE & EBP CHARACTERISTICS*

ELEMENT	Conceptual Definitions	Related Observations/Tips	Measurement
**EVIDENCE & EBP CHARACTERISTICS**	***Evidence* =** Specified sources of information relevant to the specific EBP, including research/published guidelines, clinical experience, patient experience, and/or local practice information. These sources have presumably been subjected to scrutiny, e.g., by the research team or a national body, and are judged to support or refute effectiveness of a targeted EBP intervention/ recommendation.<br><br>***EBP Characteristics*** = Attributes describing the nature of the implementable form of the evidence/ practice recommendation.	▪ As "evidence" is socially constructed [4], the perceptions of targeted stakeholders regarding the nature and quality of these varying sources of evidence are key to development of an implementation strategy. This includes perception of the form of the evidence-based clinical recommendation/intervention; i.e., the recommended practice as a guideline, policy, procedure, protocol, program, optional clinical reminder, decision algorithm, etc. At times such transformed findings/evidence is supplemented with additional content based on the judgment or consensus of its creator (e.g., consider the mixed nature of various guidelines or protocols)<br><br>▪ It is critical that researchers recognize the following:<br>o Aspects of the EBP that are core or central to its influence on outcomes and thus must be maintained in any guideline or other form of the implementable recommendation<br>o Elements that can be tailored to enhance change at a given site.<br>o Strength of the evidence underlying its implementable form<br><br>▪ Perceptions of key stakeholders can be influenced by various attributes [7,33] related to this EBP and its evidentiary source/s.	▪ Two quantitative measurement instruments have been developed that incorporate major components of PARIHS related to *Evidence*: The Organizational Readiness to Change Assessment (ORCA) [18] and a survey developed by Bahtsevani and colleagues [35].<br><br>▪ Sample qualitative diagnostic questions for use in task-oriented projects are listed below and are for the most part based on adaptation of items from the Kitson et al. Appendix related to *Evidence* [5]. This 2008 Appendix is said to outline "diagnostic and evaluative measures," but it is not a formal "tool."<br><br>o Initial, diagnostic evaluation is herein referenced as the first stage of an implementation project's formative evaluation [20].

EVIDENCE REFERENCE TOOL: Definitions for a "Revised" *EVIDENCE* Element: *EVIDENCE & EBP CHARACTERISTICS*

Related *Sub-Elements*	Conceptual Definitions	Detailed Observations/Tips regarding Sub-elements	Sample, Optional Questions to Guide Formative Evaluation (FE)
- *Research and published guidelines*	Findings from various types of studies using quantitative, qualitative or mixed method designs, as well as published guideline recommendations.	• Knowledge re: the following is important for the study team and their diagnostic & intervention planning work:<br><br>- The strength of this type of evidence (SoE) underlying the EBP intervention, including any *controversies* regarding its strength/credibility and any *qualifiers* for different subsets of patients.<br>• The latter might suggest "appropriate variations" as part of the practice recommendations [25,26].<br><br>- "Evidence" is usually, but not always, seen as more persuasive if there is published research evidence and if there is an absence of controversy about that evidence.	- To what extent are stakeholders aware or knowledgeable regarding this evidence?<br><br>- To what extent is the research evidence re: this EBP perceived as being of sufficiently high quality?<br><br>- To what extent is there consensus among colleagues about the applicability of this research to their situation?<br><br>- To what extent do key stakeholders value research vs. local knowledge and opinion?<br><br>- To what extent are there any controversies about the strength of the evidence?<br><br>- To what extent are there any qualifiers from research or perceived by stakeholders to its application?

EVIDENCE REFERENCE TOOL: Definitions for a "Revised" *EVIDENCE* Element: *EVIDENCE & EBP CHARACTERISTICS*

Related *Sub-Elements*	Conceptual Definition	Detailed Observations/Tips regarding Sub-elements	Sample, Optional Questions to Guide Formative Evaluation
- *Clinical experience*	Knowledge "expressed and embedded in practice," which is "often tacit and intuitive." Can be derived from the experience of others in addition to personal practical knowledge. This type of knowledge needs to be made explicit for it to be disseminated, critiqued, and developed [4]	• *Clinical experience* comes into play in implementation as a lens through which the recommended EBP will be viewed/judged: i.e., it is played out in terms of stakeholders' own "experience" with the targeted practice and related values, beliefs, history with change, and related motivations for change:<br>- Perceptions regarding clinical experience may or may not be based upon accurate information or cumulative facts<br>- *Evidence* is usually seen as more persuasive if published research evidence matches evidence from clinical experience.<br><br>• *Clinical experience* can be assessed through:<br>- key opinion leader views<br>- formal clinical leader views<br>- focus groups<br><br>• "Strength of evidence" in this case refers to the need to obtain affirmation or direct information re: the degree of consensus among critical stakeholders, rather than relying upon the perception of isolated, individual clinicians — unless there are individuals accurately identified as key opinion leaders.<br><br>• There is a need to be sensitive to "whose experience" has influence for this EBP and who is/needs to be in consensus (e.g., practicing clinicians and/or clinical leaders). Lack of consensus at or within any critical level or group can of course be a show-stopper.	- What is the strength and nature of motivation for/against the EBP?<br><br>- To what extent do opinion leaders in the "unit" support this EBP?<br><br>- To what extent is there (does there need to be) consensus among clinicians (from which groups/disciplines) or other key stakeholders about this EBP?<br><br>- To what extent do members of the targeted audience see congruence between the EBP/change and previously adopted changes?<br>• Was that a positive or negative "experience"?

EVIDENCE REFERENCE TOOL: **Definitions for a "Revised"** ***EVIDENCE*** **Element:** ***EVIDENCE & EBP CHARACTERISTICS***

**Related *Sub-Elements***	**Conceptual Definition**	**Detailed Observations/Tips regarding Sub-elements**	**Sample, Optional Questions to Guide Formative Evaluation**
- *Patient experiences, needs and preferences*	Various forms of patient-related information relevant to implementation and intervention planning for a targeted EBP, e.g.:<br>- Patients' previous experiences of related care<br>- "Patients' knowledge of themselves, their bodies and social lives." [4]<br>- Patient preferences, which may or may not be based upon accurate research evidence<br>- Patient stories/narratives/recorded perceptions:	• Not all EBPs may directly involve patients, or their families/significant others. Therefore, the relevance of patient evidence to the EBP should be assessed (e.g., clinicians may be queried in a general focus group on the project re: any previously unidentified side effects or pressure from patients for a new treatment.).<br>• Affirmed information regarding these issues may come from the literature on the relevant patient population; or, if feasible and applicable, could also s come from local evidence.<br>• Information about this sub-element can help implementers buffer and address patient concerns or related barriers; e.g., it could provide relevant approaches to guide providers in tailoring an EBP to meet differing patient needs, address various beliefs, or overcome related barriers.<br>• Patient preferences are also important because use of such evidence is consistent with a general value to deliver *patient-centered care*.	- What do we know/need to know about patient experience with this EBP?<br>- How do different patients experience the EBP (e.g., women v. men; minorities v. white)?<br>- To what degree is the EBP congruent with patient preferences?<br>- Do patients see the gap or suggested change as a big problem for themselves?<br>- To what extent are stakeholders aware of this type of evidence?<br>- To what extent does this unit/facility value patient input and participation?<br>o In principle<br>o In actions

EVIDENCE REFERENCE TOOL: Definitions for a "Revised" *EVIDENCE* Element: *EVIDENCE & EBP CHARACTERISTICS*

**Related *Sub-Elements***	**Conceptual Definition**	**Detailed Observations/Tips regarding Sub-elements**	**Sample, Optional Questions to Guide Formative Evaluation**
- *Local practice information*	Data re: study/project sites, providers, and practices directly related to the targeted EBP, which currently exist or are needed by the implementation team. This includes, for example, the following types of information/data:<br>▪ Current practice gaps and outcomes<br>▪ Peer review data or report cards<br>▪ QI or program evaluation data<br>▪ Local barriers or facilitators re: similar change projects<br>▪ Financial implications<br>▪ Patient perceptions and experiences<br>▪ Other survey reports	• It is important to understand stakeholders' current awareness of existing data and related tools, both within and external to the sites; e.g., re:<br>- Audit and performance data, as regarding current practice gaps or outcomes<br>- Data related to the local context/work environment, e.g., from routine organizational surveys<br>• If current data do not exist locally or within a broader related healthcare system, then determine what critical data need to be collected at the local level, particularly in relation to long-term sustainability needs, for example<br>- Practice adherence data, as well as related barriers<br>- Outcome indicator levels in relation to the initial gap<br>• Be judicious and, based on the team's knowledge of the type and characteristics of such an EBP, decide what is absolutely a *need* vs. *nice* to know for each practice setting	- To what extent are stakeholders aware of local data re: their actual performance?<br>- To what extent does available evidence regarding related performance fit with stakeholder/s' understanding and beliefs about the issue?<br>- To what extent are the desired data re: this EBP existent and routinely accessible either at the various sites or at another level?<br>- What are the specific types of locally collected data not currently available that need to be collected re: local practice and context?<br>- Is it feasible {with initial assistance of the study team, as needed} to collect needed data at appropriate levels re: the targeted practice?<br>- To what extent do stakeholders believe local data accurately reflect their actual performance?

EVIDENCE REFERENCE TOOL: Definitions for a "Revised" *EVIDENCE* Element: *EVIDENCE & EBP CHARACTERISTICS*

Related *Sub-Elements*	Conceptual Definition	Detailed Observations/Tips regarding Sub-elements	Sample, Optional Questions to Guide Formative Evaluation
- *Other characteristics of the targeted EBP*	Attributes or designated features of an EBP that may influence the likelihood of its adoption	• Knowledge of these attributes, based on a reflective analysis by the team and diagnostic data, is important to creation or refinement of an implementation plan.<br><br>• Key attributes to consider are as follows [7,33]:<br><br>- *Relative advantage*: Stakeholders' perception of the advantage [or disadvantage] of implementing the EBP versus an alternative solution; or stakeholders' cost-benefit perceptions compared to other alternatives.<br>• If targeted adopters believe in the benefit/s of implementing an EBP, the likelihood of adoption is increased. In addition, benefits should ideally outweigh other potential/competing responsibilities.<br><br>- *Observability:* Potential adopters' ability to see the EBP being used in practice, as through a pilot study, demonstration unit or role model.<br><br>- *Compatibility*: The degree of tangible fit between the nature of an EBP and the meaning and values attached to it by individuals who are tasked with its implementation; or how an EBP aligns with individuals' own norms, values, and perceived risks and needs; and how an EBP fits with existing workflows and systems. [See *Context* re: fit with broader issue of organizational priorities.]	Consider the following, relative to the applicable attribute:<br><br>*Relative advantage*:<br>- To what extent do key stakeholders believe the targeted EBP will improve practice/outcomes?<br>- To what extent do they see a need for this EBP? (*This is related to "tension for change."*)<br>o To what extent will the EBP meet (or not meet) that need?<br>- To what extent is the targeted behavior seen by stakeholders as an essential, core part of their practice?<br><br>*Observability:*<br>- To what extent will results or usefulness be easily observable to users?<br><br>*Compatibility:*<br>- How will the EBP affect current work processes and roles for adopters and other services/stakeholders?<br>o In particular, changes that affect individuals' status, authority, access to resources, or their responsibility, duties, burden, etc.<br>- How do targeted clinicians perceive the fit between the EBP and current unit/organizational priorities?<br>- To what extent do members of the targeted audience see congruence between the EBP/change and relevant values, beliefs and unit norms?<br>- To what extent do members of the targeted audience see congruence between the EBP/change and previously adopted changes/ideas?<br>- To what extent do members of the targeted audience believe the EBP/change can address an identified problem?<br>- How similar is the new EBP to existing services/practices?

EVIDENCE REFERENCE TOOL: Definitions for a "Revised" *EVIDENCE* Element: *EVIDENCE & EBP CHARACTERISTICS*

- *Other characteristics of the targeted EBP* (Continued)		- *Complexity*: Perceived feasibility and difficulty of implementation, reflected by duration, scope, radicalness, disruptiveness, centrality, intricacy and number of steps required to implement.	*Complexity:*<br>- How easy or difficult is the EBP/change to understand and use…for whom?<br>- To what extent can the EBP/change be easily described or communicated to others?<br>- How hard was/is/will it be to implement?<br>- To what extent do targeted clinicians have needed skills?<br>- How complex do stakeholders believe implementation would be for the users and/or for the system to support/enable the change?<br>o E.g., would it require internal funds or a new role? Would it impact other operations negatively?<br>- How easy or complex is it to adapt internal processes or systems to enable implementation of the EBP … without losing the core/essence of the *evidence-based practice*?<br>- How complex may it be for the users to sustain the EBP as other new changes come on line?<br>- How complex will it be for the system to sustain/spread the change?
		- *Trialability:* The ability to test the EBP on a small scale in the organization, and to be able to reverse course (undo implementation) if warranted.	*Trialability*:<br>- Can the EBP be "piloted" or "demonstrated" to be useful/successful? If major resistance exists, are there stakeholders open to a pilot, as an eventual form of persuasion or marketing?<br>- To what extent can this EBP/change be tried out on a limited basis…either by individuals or a selected, influential subset?
		- *Design quality and packaging:* Perceived excellence in how the EBP is bundled, presented, and assembled.	*Design quality and packaging*<br>- What is the perceived quality of training, materials, equipment, and support?
		- *Costs:* Of the EBP intervention and those associated with its implementation, including investment, supply, and opportunity costs.	*Cost:*<br>- What are the actual and opportunity costs for initial and on-going implementation of the EBP?<br>- Can a persuasive business case be made for the change?
