# Supplementary material for: A Guide for applying a revised version of the PARIHS framework for implementation
Source: Implement Sci. 2011 Aug 30;6:99. doi: 10.1186/1748-5908-6-99 (PMC3184083; doi:10.1186/1748-5908-6-99)
Supplement: Additional file 2 — CONTEXT REFERENCE TOOL: Definitions for a "Revised" PARIHS "Context" Element: Contextual Readiness for Targeted EBP Implementation. This Context Reference Tool provides explicit definitions for this element and its sub-elements; related, detailed explanations and observations; and sample, optional questions to guide formative evaluation. [file 1748-5908-6-99-S2.PDF]

CONTEXT REFERENCE TOOL: Definitions for a "Revised" PARIHS "*CONTEXT*" Element: *CONTEXTUAL READINESS FOR TARGETED EBP IMPLEMENTATION*

A "Revised PARIHS Framework": Elements & Sub-Elements for a Task-Oriented Approach to Implementation: SI = f (E,C,F)

E: *Evidence and EBP Characteristics:*

- *Research and published guidelines*
- *Clinical experiences and perceptions*
- *Patient experiences, needs and preferences*
- *Local practice information*
- *Characteristics of the targeted EBP*

<table>
<tr>
<td>C: Contextual Readiness for Targeted EBP Implementation
- Leadership support
- Culture
- Evaluation capabilities
- Receptivity to the targeted innovation/change</td>
<td>Reference Tool Content
Information in this and the other tools in this Revised PARIHS Guide can be used to prepare a proposal, including related methodology, and follow-up reports. More specifically, this Context tool can be used to:

• Think more specifically about the nature of Context and enhance communication of that understanding to reviewers and other readers.
• Identify potential Contextual barriers that may need to be better understood and /or addressed in the implementation strategy (e.g., thinking through the type of leadership support that will be needed given the type of innovation to be implemented).
• Identify diagnostic/evaluative questions for a semi-structured interview relevant to the need to understand selected aspects of the Context, applicable to this specific EBP change.
• Develop and organize a retrospective interpretive evaluation [20] to explore the perceived influence of Contextual features on implementation of the targeted EBP.

NOTE: In all cases, the list of multiple items should be considered an optional menu from which to choose components of prime relevance to implementation of the targeted EBP.</td>
</tr>
</table>

F: *Facilitation*

- *Role of facilitator:*
  - *Purpose, external and/or internal role*
  - *Expectations and activities*
  - *Skills and attributes of facilitator*
- *Other implementation interventions identified through site diagnostic assessment and relevant sources*

SI: *Successful Implementation*

- *Implementation plan and its realization*
- *EBP innovation uptake: i.e., of clinical interventions and/or delivery system interventions*
- *Patient and organizational outcomes achievement*

CONTEXT REFERENCE TOOL: Definitions for a "Revised" PARIHS "*CONTEXT*" Element: *CONTEXTUAL READINESS FOR TARGETED EBP IMPLEMENTATION*

ELEMENT	Conceptual Definitions	Related Observations/Tips	Measurement
CONTEXTUAL READINESS for Targeted EBP IMPLEMENTATION	***Context*** = The environment or setting in which the proposed evidence-based change is to be implemented:<br>• "…physical environment in which practice takes place…. [with] boundaries and structures that together shape the [setting] for practice*"*[2]<br>• "...includes the environmental characteristics surrounding the implementation effort"[7]<br><br>***Readiness for implementation*** = Willingness, preparedness and capacity for implementation of a specific, discrete practice change	▪ The overall element has been renamed from simply "Context" to a title in part related to wording in Kitson et al.'s Appendix (2008) [5].<br><br>▪ The content of its sub-elements now reflects a focus on a *specific* implementation of a *targeted* EBP.	▪ Four quantitative measurement instruments have been developed that incorporate aspects of PARIHS related to *Context*:<br>- The Organizational Readiness to Change Assessment (ORCA) tool from Helfrich et al, 2009 [18]<br>- A survey developed by Bahtsevani and colleagues, 2008 [35]<br>- A Context Assessment Instrument (CAI) developed by McCormack et a., 2009[12]<br>- The ACT (Alberta Context Tool) from Estabrooks et al. 2009[1]<br><br>▪ Sample qualitative diagnostic questions for use in task-oriented implementation projects are listed below and are for the most part based on adaptation of items from the Kitson et al. (2008) Appendix [5] related to *Context*. This 2008 Appendix is said to outline "diagnostic and evaluative measures," but it is not a formal "tool."<br><br>o Initial, diagnostic evaluation is herein referenced as the first stage of an implementation project's formative evaluation [20].

[1] Estabrooks CA, Squires JE, Cummings GG, Birdsell JM, Norton PG. (2009). Development and assessment of the Alberta Context Tool. BMC Health Serv Res Dec 15;9:234.

CONTEXT REFERENCE TOOL: Definitions for a "Revised" PARIHS "*CONTEXT*" Element: *CONTEXTUAL READINESS FOR TARGETED EBP IMPLEMENTATION*

Related Sub-Elements	**Conceptual Definitions**	**Detailed Observations/Tips regarding Sub-elements**	**Sample, Optional Questions to Guide Formative Evaluation (FE)**
- *Leadership support*	***Leadership* =** Individuals in designated positions "…at any level of the organization including executive leaders, middle management, front-line supervisors, and team leaders, who have a direct or indirect influence on the implementation." [7]<br><br>***Leadership Support*** = Behaviors, attitudes and actions of leaders that reflect readiness or receptivity to a change. [17]	• This interpretation of leadership focuses primarily and directly on the *support* of relevant leadership, at applicable levels, for implementation and sustainability of a targeted EBP. This may include, depending upon the EBP, a setting administrator, clinical director of a unit, nurse manager, medical leader of a specialty, etc.<br>o It is possible that there are leaders external to the targeted site/s whose support is also critical.<br><br>• Relevant components under this sub-element focus on observable behaviors, attitudes, and decisions of key leaders.<br><br>• In general, relevant leaders' "supportive" *actions* can be characterized by various types of managerial behaviors or responsibilities, within a change/innovation situation such as EBP, as listed below. These are not directly taken from the basic PARIHS framework but rather have been adapted based on the following: i.e., a task-oriented view of related PARIHS sub-elements; supplemental information from relevant publications [17,36-37]; relevant EBP behaviors of transformational leaders [17]; and an effort to use language more familiar to targeted researchers:<br><br>o *Clarify roles, e.g.*, ensuring transparency regarding both *project*-related and relevant *change*-related role responsibilities and accountabilities.<br>o *Marketing, e.g.*, indicating enthusiasm/support for/value of/need *for the targeted change* and, as appropriate, its "fit" with organizational priorities/needs. Includes visibility of personal support through related behaviors over time….and not merely in the beginning of a project [23]<br>o *Communicating*, e.g., overseeing the provision of adequate and timely information re: *the change* to all relevant stakeholders; and providing a channel for input/feedback from those being affected	NOTE:<br>1. Although still relevant to primarily a task-oriented implementation project, some of the following optional questions are focused on general leadership behaviors and not on a targeted/type of change. Applicability may depend on the timing of/stage of FE; e.g., pre- versus post-implementation.<br>2. The level or levels of "leader" under assessment should be clearly identified.<br><br>- To what extent do leaders show active and visible support for this change or this type of EBP and implementation?<br>o Is the leader willing to engage with the study team for planning?<br>o Is the leader willing to provide connections/entrees for the study team?<br>o Does the leader have experience/comfort in this role?<br>o Does the leader hold service directors accountable for collaboration and coordination in such change efforts/this change effort?<br><br>- To what extent are appropriate stakeholders or teams held accountable and incentivized or rewarded to carry out the implementation?<br>o What about past experiences with this *type* of change?<br><br>- To what extent does a leader indicate the willingness to and in fact does this leader communicate the priority of this implementation?<br><br>- To what extent does the leader use existent communication channels to keep staff up to date about implementation progress?<br><br>- To what extent does the leader obtain feedback and input re: the change?<br><br>- To what extent does the leader use existent communication channels to obtain feedback and input re: the change?

- *Leadership support (continued)*		o *Enabling effective teamwork, e.g.*, creating or identifying teams of appropriate individuals needed to help *implement the project;* and in turn providing them with needed team leadership, decision-making authority, expertise and resources.<br>o *Enhancing collaboration, e.g.*, communicating to *involved* units or departments the *expectations* regarding cross-service/discipline teamwork and responsive support<br>o *Providing general support, e.g.*, being available for problem resolution to those charged with the change; helping resolve barriers; giving positive feedback and other incentives for *the change;* ensuring the existence or availability of *needed* systems, structures and resources for implementation and sustainability [24]<br>o *Ensuring accountability, e.g., holding* those charged with *the change* accountable for progress and outcomes	- To what extent is a clear and appropriate leader designated, authorized, and enabled to create/lead an implementation project team:<br>o Is free time feasible or made possible for that leader?<br>o Is a mentor available if needed by the project leader??<br>o Are adequate instructions being provided, including member and team leader expectations?<br>o Does the authorizing agent empower the leader to do the task?<br><br>- To what extent is the appointment of requested champions or opinion leaders adequately addressed, in terms of:<br>o Availability<br>o Credibility<br>o Clarity of the role<br><br>- To what extent are appropriate staff and time provided for an<br>- implementation team:<br>o In terms of availability<br>o In terms of credibility<br>o In terms of representing key stakeholder groups<br><br>- To what extent are needed resources, access to key stakeholders, space requirements, etc. committed and provided in a timely manner?<br><br>- To what extent do key leaders see this EBP as a priority at this point in time?<br><br>- To what extent is the leader aware of or scanning for barriers to implementation?<br><br>To what extent is the leader responsive to requests for support to eliminate barriers?

CONTEXT REFERENCE TOOL: Definitions for a "Revised" PARIHS "*CONTEXT*" Element: *CONTEXTUAL READINESS FOR TARGETED EBP IMPLEMENTATION*

Related Sub-Elements	Conceptual Definition	Detailed Observations/Tips regarding Sub-elements	Sample, Optional Questions to Guide Formative Evaluation (FE)
- *Culture*	***Cultu**re* = "the way things are done around here"; or "prevailing values and beliefs as a prerequisite to introducing and sustaining change"[10]<br><br>o Also = the way people *believe* things are or can be done around here, whether it is accurate or not.	• The implementation team must be clear as to what the relevant unit/facility/service is for the EBP and what, if any, aspect of culture as defined therein may be applicable to the targeted EBP:<br>o Caution: There can be different cultures on different units/services and even among different disciplines. The goal is to understand only *critical* cultural issues and, if possible, aspects that either:<br>- Are mutable [which is difficult as culture does not change easily or overnight]<br>- Provide insight for buffering resistance and enhancing needed behaviors.<br><br>• Task-oriented implementation is usually not about changing an existent culture in a major way, but about understanding how to enhance change within the identified critical aspects of the culture OR about working within or around aspects of a culture. This might require assessment of the perceptions/attitudes of key stakeholders regarding the following types of topics:<br>o The need for collaboration and team work across disciplines<br>o The assumed feasibility of EBP on this unit/organization, specifically regarding this type of change or perhaps any change<br>o Autonomy [and whether one …such as a given discipline or role…should have it or not relative to the targeted EBP]<br>o Communication or routine information updates [and whether one or various groups should get such information or not] re: this type of change<br>o Participation or input to decisions [and whether one or various groups should have it or not] relative to this type of change<br>o The nature of a discipline's professional role [rights and privileges and expected scope of responsibilities]<br>o The value of data-based decision-making or EBP	- To what extent do key stakeholders believe their unit/service/organization can implement this type of EBP?<br>o Check perceptions re: similar implementation efforts in the past<br><br>- To what extent do key stakeholders on/at the unit/site usually collaborate across professions, disciplines, units, specialties, departments, etc?<br>o Check examples of successful collaboration<br>o Check examples of unsuccessful collaboration<br><br>- To what extent is there usually effective collaboration within teams at the site?<br>o Established teams<br>o Newly formed teams for this type of change project<br><br>- To what extent does this organization/unit/facility/etc value open communication, dialogue and staff input?<br>o Have, and if so how have, key stakeholders been involved in determining whether/how this initiative is going to be implemented?<br><br>- To what extent do key stakeholders see targeted changes as appropriate/inappropriate to their role?<br>o Is the targeted provider function seen as core or peripheral to the providers' view of their role: e.g., do they care 'who' does or has to do this? Who should "own/control" this practice?

Related Sub-Elements	Conceptual Definition	Detailed Observations/Tips regarding Sub-elements	Sample, Optional Questions to Guide Formative Evaluation (FE)
- *Evaluation capabilities*	***Evaluation*** capabilities = Characterized herein as the following:<br><br>• The existence of systems/processes for collecting and using EBP–related data within an implementation site [Note: such systems may be available to the site through external repositories]<br>• The potential of relevant systems within targeted sites to produce needed data now and, for sustainability, in the future<br>• The ability of relevant systems within targeted sites to make the above data available to key stakeholders in meaningful forms	• Systems may already be in operation and thus have "capability" to provide data relevant to the targeted EBP or are routinely collecting relevant local data. Ideally, such data are being collected from multiple sources via various site departments or external offices; e.g., from quality; finance, special projects, education departments, or externally:<br>o Data from multiple sources and from use of multiple methods are more likely to provide a richer understanding of related topics (e.g., use of quantitative and qualitative methods) [2,3].<br>• Note the dynamic relationship between this sub-element and Local Practice *Evidence*: i.e., the issue here is whether the site has needed *systems and processes* to collect AND disseminate useful and needed types of data in general; and in this case whether systems are available for this project in the timeframe needed.<br>o The Local Practice Information sub-element relates to the actual existence, availability and awareness of stakeholders re: relevant data for this EBP<br>• Certain characteristics of local data and its communication should be considered, relative to its use for targeted implementation:<br>o Perceived usefulness and credibility to stakeholders<br>o Timeliness and feasibility for sustainability of measurement, e.g., in terms of resources<br>o Coverage, in terms of data summary relative to:<br>- Multiple key stakeholders<br>- Relevant levels; e.g., providers; patients; managers at different levels; teams; etc<br>• The capability of local structures, systems, or processes to routinely collect needed local data is critical for sustainability. Related resources (such as finances or Information Technology [IT] departments) and expertise, as well as key leadership support are obviously relevant factors; and all are dynamically reflected in other *Contextual Readiness* sub-elements.	- To what extent are needed diagnostic/ baseline data available for targeted sites?<br>o Routinely?<br><br>- To what extent are targeted sites capable of producing needed baseline, other diagnostic or sustainability data?<br><br>- To what extent does the site routinely make available information upon which decisions related to such an EBP are made?<br>o Are they capable [e.g., in terms of resources and experts] of generating such reports?<br>o Are they capable of generating information through a variety of methods (e.g., anecdotal stories, feedback reports, statistical displays)?<br>o How credible, timely, useful, etc. are such reports/communications perceived to be?<br><br>- To what extent do sites routinely collect (and use) evaluative data on change programs?<br>o Does this include FE?<br>o Do they have resident evaluative expertise?<br><br>- Do the sites have needed resources (e.g., IT or information technology) and related expertise to obtain targeted local data?

CONTEXT REFERENCE TOOL: Definitions for a "Revised" PARIHS "*CONTEXT*" Element: *CONTEXTUAL READINESS FOR TARGETED EBP IMPLEMENTATION*

Related Sub-Elements	**Conceptual Definitions**	**Detailed Observations/Tips regarding Sub-elements**	**Sample, Optional Questions to Guide Formative Evaluation (FE)**
Receptivity to the targeted EBP/change	***Receptivity*** = Readiness or fit of critical features of the environment as they specifically relate to a targeted EBP.	• Critical features of readiness to be explored are as follows:<br>o *Resources*, including human (e.g., staff, experts and champions), hardware, software, financial, etc. This would include the existence of individuals capable of performing various change agent roles<br><br>o *Space, physical locations and related equipment*<br><br>o *Decision-making authority*, i.e., power and authority, related to the EBP at multiple levels<br><br>• *Fit* of the EBP innovation with current strategic organizational intent and goals, including whether it is critical for practice/patient care	- To what extent does the physical location, configuration, etc meet the needs of the EBP/change?<br><br>- To what extent do communication channels or formal networks exist to routinely keep staff up to date about organizational happenings, such as implementation progress?<br><br>- To what extent do communication channels or formal networks exist for staff to obtain feedback and provide input re: key topics, such as the targeted change?<br><br>- To what extent are outside networks available (professional, peer, other) that may be important for or potentially facilitative to implementing the EBP?<br><br>o To what extent do key stakeholders have connections with or access to outside professional/peer/other networks that might enhance or support implementation of the EBP? (Which stakeholders, at which levels of authority?)<br><br>- To what extent do key stakeholders have the power and authority to carry out expected tasks of change, such as this EBP — during the adoption process and routinely?<br><br>- To what extent are appropriate *internal facilitation* resources available as needed for an EBP implementation, such as the following:<br>o Individuals with needed skills and attributes to assume the role of an internal "facilitator"<br>o Individuals with needed skills and attributes to mentor internal "facilitators"?<br><br>- To what extent are other sufficient resources (e.g., personnel, personnel with allocated time, equipment, finances, IT support, and clinical experts) available as needed for the EBP?<br>- To what extent are training resources available for education related to change and its leadership (Trainers; Experts; Consultants)?<br><br>- What are current organizational priorities?<br>o To what extent does the EBP appear to fit with those priorities?<br>o What is the current level and timing of attention on other priorities that might interfere with implementing this EBP?
