# Supplementary material for: A Guide for applying a revised version of the PARIHS framework for implementation
Source: Implement Sci. 2011 Aug 30;6:99. doi: 10.1186/1748-5908-6-99 (PMC3184083; doi:10.1186/1748-5908-6-99)
Supplement: Additional file 3 — FACILITATION REFERENCE TOOL: Definitions for a "Revised" PARIHS FACILITATION Element. This Facilitation Reference Tool provides explicit definitions for this element and its sub-elements; related, detailed explanations and observations; and sample, optional questions to guide planning the critical details of implementation. [file 1748-5908-6-99-S3.PDF]

FACILITATION REFERENCE TOOL: Definitions for a "Revised" PARIHS *FACILITATION* Element

A "Revised PARIHS Framework": Elements & Sub-Elements for a Task-Oriented Approach to Implementation: SI = f (E,C,F)

E: *Evidence and EBP Characteristics:*

- *Research and published guidelines*
- *Clinical experiences and perceptions*
- *Patient experiences, needs and preferences*
- *Local practice information*
- *Characteristics of the targeted EBP*

C: *Contextual Readiness for Targeted EBP Implementation*

- *Leadership support*
- *Culture*
- *Evaluation capabilities*
- *Receptivity to the targeted innovation/change*

<table>
<tr>
<td>
F: Facilitation:

Role of facilitator:

Purpose, external and/or internal
Expectations & activities
Skills & attributes of facilitator

Other implementation interventions

Related to "E"
Related to "C"
Other

</td>
<td>
Reference Content
Information in this and the other tools in this Revised PARIHS Guide can be used to prepare a proposal, including related methodology, and needed reports. More specifically, this Facilitation tool can be used in the following ways, i.e., to:

Think more broadly about the nature of Facilitation
Clarify the team's understanding of the Facilitation element and its planning
Enhance communication of that understanding to reviewers and other readers
Think through and determine the types and specifics of facilitator roles needed for various sites, as well as other change needs and related project team activities
Help construct and tailor appropriate implementation interventions beyond the facilitator role, in part through use of a "tickler list" of potential implementation issues and interventions
Guide the planning of critical details of implementation through a set of potential planning questions

NOTE: In all cases, the list of multiple items should be considered an optional menu from which to choose components of prime relevance to implementation of the targeted EBP.
</td>
</tr>
</table>

SI: *Successful Implementation*

- *Implementation plan and its realization*
- *EBP innovation uptake: i.e., of clinical interventions and/or delivery system interventions*
- *Patient and organizational outcomes achievement*

ELEMENT	Conceptual Definition	Related Observations/Tips	Measurement
FACILITATION:	***Facilitation* =** A process of "helping individuals and teams to understand what they need to change and how they need to change it in order to apply evidence to practice" [2]<br><br>*Facilitation* = A deliberate process of interactive problem solving and support that occurs in the context of a recognized need for improvement and a supportive interpersonal relationship [19]<br><br>• In this altered version of PARIHS, the *Facilitation* element contains two sub-elements, both of which reflect implementation interventions:<br>1. The *role* of a facilitator<br>2. Other *implementation* interventions that the facilitator/s and other members of the implementation/study team use to enhance implementation	• There are two major purposes of Facilitation:<br>- "Task"-focused, which provides help and support to achieve a specific goal<br>- "Holistic"-focused, which helps individuals and teams analyze, reflect and change *their own* attitudes, behaviors and ways of working, related to a planned change [5]<br><br>• This *Guide* emphasizes task-oriented *implementation*, rather than organizational *transformation*. It therefore emphasizes task-focused *Facilitation* and thus support for implementation of targeted EBPs, rather than a more holistic process of *Facilitation* such as 'Critical companionship' [5].<br><br>• "The key to 'appropriate' facilitation is matching the purpose, role, and skills to the needs of the situation" [2]. With our expanded conceptualization of *Facilitation*, *appropriateness* would extend to selection of additional implementation interventions that are part of the implementation plan and that are matched to the needs of the situation.<br><br>• The two sub-elements of *Facilitation* are inextricably linked; i.e., diagnostic analysis [DA] of issues/needs will help to determine all types of implementation interventions, including the need for an internal and/or external facilitator. DA will thus help to flesh out the specific type, purpose, expectations, activities and skills needed for a given Facilitator role; and these in part will be defined in relation to other types of implementation interventions required.<br><br>• It is important to note that our modifications to PARIHS focus on a task-oriented AND team-based approach to implementation. The facilitator is not the only individual who assesses/diagnoses targeted sites through formative evaluation (FE) methods; nor the only individual who develops the "other implementation interventions"; nor the only individual to implement or reinforce the "other implementation interventions."<br>- Various researchers try to establish partnerships with implementation facilities/settings and engage their specific support for adoption, which may or may not involve an internal implementation facilitator or team.	▪ Two quantitative measurement instruments have been developed that incorporate components of PARIHS related to *Facilitation*: i.e., the Organizational Readiness to Change (ORCA) tool from Helfrich et al, 2009 [20] and a survey developed by Bahtsevani and colleagues in 2008 [35]:<br>- ORCA measures the *capacity* of an organization for internal facilitation.<br>- The Bahtsevani tool has two items on F, characterized by the authors as relating to the "context of care."<br><br>▪ The 2008 PARIHS Appendix did not elaborate upon planning for *Facilitation* [5].For this revised framework, we have included questions that focus on a set of *planning* issues that the project team might need to address, based on the diagnosis of E and C. These planning questions address both different types of facilitator roles and other implementation intervention needs.

		• A facilitator is not the only individual to "help" individuals/teams or support them during implementation. The supportive behaviors of leadership within the facility, as described under *Context*, are a form of "help." However, most such leaders are not given the responsibility to fulfill the purpose and role expectations described for an appointed *facilitator*.<br>- IF a formal leader, such as a nurse manager or clinical nurse specialist, is given or inherently assumes the responsibility of a "Facilitator," as defined in the PARIHS framework, then emphasis should be on them as a Facilitator versus "*leadership."*<br>- At the same time, it will be essential that formative evaluation (FE) [20] be used to assess key behaviors that made a difference in SI; and be used to assess whether the inherent power and authority of a concurrent, contextual-based leadership role made a difference—including:<br>o ability to obtain "*Leadership support"* from others to remove contextual barriers beyond that manager/leader's scope<br>o ability to meaningfully hold staff accountable for adherence.	

Related Sub-Elements	**Conceptual Definitions**	**Detailed Observations/Tips regarding Sub-elements**	**Sample, Optional Questions to Guide Project Planning**
Role of Facilitator<br><br>(NOTE: The content regarding this sub-element focuses primarily on an external facilitator.)	*Facilitator* = An individual assigned to an explicit role; and said role is an implementation intervention.<br><br>• A facilitator is an explicit, appointed *change agent "role"* and encompasses the behaviors and characteristics of individuals assigned to that role. More specifically, it is [2] :<br>o "…an appointed role as opposed to that of, for example, an opinion leader who acts as a change agent through his/her own personal reputation and influence."<br>o "…an appointed activity played by an individual."<br>o An "…individual enacting a specified purpose relative to implementation, employing a broad spectrum of mechanisms"	• Facilitation, as enacted by a facilitator role "….is a process that depends upon the person (the facilitator) carrying out the role with the appropriate skills, personal attributes and knowledge" [5].<br><br>• The following terms may be used by various projects to reference a full or part-time facilitator. However, caution must be taken as to the intent vs. reality of the role expected of individuals assigned within a facility as, for example, a "team leader" or "champion." Bottom-line; the study team must be clear what implementation role components appear to be needed in what type of *Context* and who or what combination of individuals will need to fulfill that "role."<br>- *External facilitator:* e.g., a research team facilitator or a health system-wide level facilitator; the latter individual might be someone who works closely with other "facilitators" in the various health system sites as a mentor/expert on change/facilitation<br>- *Internal facilitator*: e.g., an individual designated within an individual site or facility for this role<br>- *Champion*: this in reality may be an appointed or emergent facilitator (part or full-time) or, conversely, may be defined in a way that does not include critical expectations for *Facilitation* [16]<br>- *Project lead or manager*: there will need to be clarity, both within a research team and within a facility, as applicable, regarding the true responsibilities and capabilities of such a role…and perhaps, the need for supplemental facilitation roles.	• Based on results of the diagnostic analysis of E & C, what type of facilitator role/s is/are needed?<br><br>o What is the contextual capacity of the project site to support an internal facilitator or other change agent?<br>o To what extent will individuals in such roles have/will be given the time **and** have the motivation to devote to needed activities [19]?<br><br>• Will the external facilitator need to address the same needs for every site; or, will a specific site's needs vary depending on their baseline characteristics?

Related Sub-Elements	**Conceptual Definitions**	**Detailed Observations/Tips regarding Sub-elements**	**Sample, Optional Questions to Guide Project Planning**
- Role purpose, external and/or internal	*Role Purpose* (in task-oriented implementation) = The facilitator's function, i.e., to guide, help and support implementation of a targeted EBP.	• In reality, this "task" orientation may require some attention to aspects of the basic PARIHS's "holistic" approach. Thus the facilitation role, overall, is best characterized as a hybrid or mixed approach that is tailored to study needs in various sites:<br>For example, an external facilitator role may need to involve a more holistic purpose when providing support for enthusiastic but unskilled internal facilitators.	• Given the types of facilitators that have been identified for the project, to what extent will a holistic approach or a mixed approach be desirable?<br>o For example, will an external or system-level facilitator be mentoring other change agents?
- Role expectations and activities	*Role expectations* = The behaviors and actions expected of the individual who has been assigned to a project facilitator position/function.<br><br>o These expected behaviors/actions are designed to:<br>- Operationalize established implementation goals for the project<br>- Directly or indirectly influence individual and process change<br>- Enhance overall implementation.	• The types of behaviors/actions listed below, expected of a facilitator, are an adaptation of PARIHS components [5] in light of author-related experiences [19]:<br>o *Regular, goal-focused contact and communications*, e.g.::<br>- maintaining structured, regular communication as well as ad hoc availability with local facilitators and others key to successful implementation<br>- providing clarity and information for internal change agents<br>- gathering local agent input on issues and concerns<br>o *Technical/practice assistance*, including linking sites to clinical or other expertise as needed<br>o *Interactive, contingent problem-solving*, from identification to resolution: e.g., JIT (just in time) education or networking, persuasive advocacy/ championing with resistant stakeholders and reviewing potential approaches/strategies for solving problems<br>o *Use of formative data ;* e.g., review of diagnostic information to understand local context; and tracking/use of implementation-focused and progress-related data [20]<br>o *Enabling, through establishing and maintaining a supportive relationship:* e.g., being available and responsive, providing interpersonal support and mentoring, and empowering local agents to act on their own<br>o *Doing for others* (short-term), perhaps as a role model; e.g., by conducting "direct implementation (intervention activities) or initiation of (problem) solutions in relation to both identified local site needs and the need to see core … intervention strategies implemented" [20] . The latter may involve interacting with various stakeholders in or outside the site.	• If the external or system facilitator will be working with internal changes agents in the local setting/s, to what extent will this facilitator need to:<br>o Guide/mentor internal change agents [how]?<br>o At least initially, "*do for others*," in terms of initiating specific *implementati*on activities?<br>• What intensity of *Facilitation* support appears to be required, again based on diagnostic information, in various sites; e.g., in terms of different degrees of goal-focused contact and communications?<br>• What system will be developed to enable reasonable, timely access to the external facilitator?<br>• To what extent is the study team prepared to support the external facilitator in problem solving efforts?<br>• What systems have been developed within the study team to regularly channel FE data to the external facilitator, team and thence to the internal facilitator?<br>• Given the nature of the change, the complexity of the implementation strategy, and the site's contextual readiness (including their contextual capacity for *Facilitation*), what amount of time must be afforded to the external facilitator's role?

		• Also relevant to the facilitator role is the degree to which role expectations are feasible within the given context. Some of the sample questions in the above right hand column therefore dynamically relate to *Context* and are included here given their immediate relevancy. Such questions could relate to internal change agent roles as well.	• What if any external networks are available to obtain resolution of issues externally, where needed, or link local sites with experts?
- Role skills and attributes	*Role skills & attributes* = The ability to perform required tasks and the possession of characteristics that enable implementation of role expectations and activities.	• In general, the nature of needed skills, attributes and characteristics will relate to the type of role expectations/activities cited above. However, it is suggested [9] that the most important "expertise could be in having the flexibility to be able to recognize the requirements of an individual situation. This may mean drawing on a combination of skills and qualities in the course of any change process."<br>o It may also require the ability and willingness to locate, and call upon, others with special skills/expertise [e.g., clinical].<br><br>• Potentially important *attributes* that the individual should be able to demonstrate to/ key stakeholders include the following [8,5,9,21]:<br>o Authenticity, realness and openness<br>o Respect and general credibility, e.g. seen to understand the evidence and change processes<br>o Accessibility, approachability, and empathy, e.g., open to being contacted, friendly and outgoing, and can establish good rapport Flexibility, e.g., ability to think laterally and non-judgmentally<br>o Responsiveness and reliability, e.g., having answers to questions or can easily find.<br>o Self confidence<br><br>• Potentially important *skills/expertise* include the following [8,9,5,19]:<br>o Communication skills, including listening, teaching, negotiating, networking and persuasion/marketing skills<br>o Implementation science expertise, including competency related to use of applicable individual and organizational change interventions and FE<br>o Organizing skills<br>o Marketing skills<br>o Subject/technical skills<br>o Problem-solving skills<br>• A facilitator does not necessarily need direct clinical expertise in the EBP area, if there are clinical experts that may be called upon. Whether facilitators need clinical experience in a relevant discipline is as yet unclear. Change-related experience may suffice, especially in health care.	- What are the implications of the following for the type of skills and characteristics needed in the external facilitator:<br>o Nature of the planned change<br>o Complexity of the implementation strategy<br>o Sites' contextual readiness, including their contextual capacity for internal facilitation<br>o Availability of technical/practice expertise among the study team, which could be accessed by the external facilitator?<br><br>- To what extent do readily available individuals for the external facilitator role actually meet the suggested attributes, skills and expertise?<br><br>- To what extent do readily available individuals for the internal facilitator role actually meet the suggested attributes, skills and expertise?<br>o What then are the internal facilitator/s' need for mentoring and support, and from whom?

Related Sub-Elements	Conceptual Definitions	Detailed Observations/Tips regarding Sub-elements and	Sample, Optional Questions to Guide Project Planning
Other implementation interventions:<br>- Related to "E"<br>- Related to "C"<br>- Other	*Implementation intervention* (beyond the facilitator role) [15]= A single method or technique to facilitate change and thereby adoption of best practice recommendations/EBP:<br>• Also referred to as an "uptake" or "adoption" or "change" intervention.	• The primary purpose of these "other" implementation interventions is also to guide, help and support uptake of a targeted EBP.<br>o Note that an EBP recommendation is termed a clinical or health promotion or delivery system intervention [15]<br><br>• "Other" implementation interventions cover the following well know types of methods; e.g.,<br>o Passive education<br>o Interactive education<br>o Audit/feedback<br>o Technological support (ECR or electronic clinical reminder or web-based programs)<br>o Engagement strategies, e.g., social marketing<br>o QI improvement collaboratives<br>o Networks<br>o Opinion leaders**<br>o Champions**<br>o Partnership contract/MoU [Memorandum of Understanding]<br>o Structure/process changes, such as roles, expectations, data systems<br>o Performance measures<br>o Targeted contextual supports [e.g., structured leadership support]<br>o Formative evaluation<br><br>[**NOTE: It is critical that clear, operational definitions be provided for these implementation interventions, in this case defined differently than facilitators.]<br><br>• The implementation plan or set of implementation interventions needed to enhance uptake of a targeted EBP is derived from multiple sources:<br>o A diagnostic assessment [FE] focused on readiness, barriers, and enablers at the targeted sites, in terms of E (see the	• Choose implementation interventions based on the results of the diagnostic analysis of E & C, e.g.:<br>o What barriers need to be addressed in various settings?<br>o What potential "facilitators" of change exist in various settings?<br>o What attributes of the EBP need to be addressed or enhanced?<br><br>• What is the level/focus/stages of change that need to be addressed, thus influencing the choice of implementation interventions, e.g.:<br>o Are you targeting individual clinicians and/or managers, a team, unit and/or multiple units?<br>o Are you targeting awareness, knowledge, motivation, attitudes, engagement, skills, behavior, processes, etc.?<br><br>• Based on findings from the literature or pilot work regarding the type of targeted EBP that is the focus of this project, which of the listed implementation interventions might be useful to enhance adoption of the desired change?

		Evidence tool) and C (see the *Contextual* tool)<br>o Information from the literature re: barriers and enablers to implementation of the type of targeted EBP/innovation relative to E and C, including previously effective implementation interventions<br>o Supplementary theories relevant to the EBP innovation<br>o Prior/pilot work of the research team<br><br>• According to Kitson et al. [5], facilitators should assess the situation, individual, team, and workplace readiness; and develop change and evaluation strategies. However, in some cases, it is a *study team* —not a lone facilitator— that develops a theory-based implementation strategy— i.e., a "bundle" of needed implementation interventions [15].	
