# Supplementary material for: A Guide for applying a revised version of the PARIHS framework for implementation
Source: Implement Sci. 2011 Aug 30;6:99. doi: 10.1186/1748-5908-6-99 (PMC3184083; doi:10.1186/1748-5908-6-99)
Supplement: Additional file 4 — SUCCESSFUL IMPLEMENTATION TOOL: Definitions for a "Revised" PARIHS Successful Implementation Element. This Successful Implementation Reference Tool provides information on three foci for evaluation of this component of the framework; related definitions and key issues; and observations and suggestions regarding relevant measurements. [file 1748-5908-6-99-S4.PDF]

(A "Revised PARIHS Framework": Elements & Sub-Elements for a Task-Oriented Approach to Implementation: SI = f (E,C,F)[1]

E: *Evidence and EBP Characteristics:*
- *Research and published guidelines*
- *Clinical experiences and perceptions*
- *Patient experiences, needs and preferences*
- *Local practice information*
- *Characteristics of the targeted EBP*

C: *Contextual Readiness for Targeted EBP Implementation*
- *Leadership support*
- *Culture*
- *Evaluation capabilities*
- *Receptivity to the targeted innovation/change*

F: *Facilitation*
- *Role of facilitator:*
  - *Purpose, external and/or internal role*
  - *Expectations and activities*
  - *Skills and attributes of facilitator*
- *Other implementation interventions identified through site diagnostic assessment and relevant sources*
  - *Related to "E"*
  - *Related to "C"*
  - *Other*

	**Reference Content**
**SI: *Successful Implementation***<br>- ***Implementation plan and its realization***<br>- ***EBP innovation uptake: i.e., of clinical interventions and/or delivery system interventions***<br>- ***Patient and organizational outcomes achievement***	Information in this and the other tools in this *Revised PARIHS Guide* can be used to prepare a proposal, including related methodology, and follow-up reports. More specifically, this *Successful Implementation* tool can be used to:<br>• Define the nature of *Successful Implementation*<br>• Clarify the team's understanding of the *Successful Implementation* element and enhance communication of that understanding to reviewers and other readers<br>• Help construct, through use of a "tickler list," an evaluation of fidelity and other critical aspects of an implementation plan's operationalization<br>• With the aid of a set of sample questions, identify needed interim and end result outcome indicators<br>• Think through the importance of and approach to evaluating use of the Revised PARIHS framework<br><br>*NOTE: In all cases, the list of multiple items should be considered an optional menu from which to choose components of prime relevance to implementation of a targeted EBP.*

[1] The Stetler et al paper [20] on formative evaluation or FE was a key basis for the SI Appendix.

Stetler, Damschroder, Helfrich and Hagedorn, A *Guide* for applying a revised version of the PARIHS framework for implementation. *Implementation Science*.

***Related Sub-Elements* (Focus of Evaluation)**	**Conceptual Definition**	**Detailed Observations/Tips regarding Sub-elements**	**Sample, Optional Questions to Guide Formative Evaluation [20]**
Implementation plan and its realization	*SI per Implementation Plan* = The degree to which implementation strategies are both fielded in the intended form/format and related adoption activities reach the targeted audience:<br><br>• For example, in a project to disseminate and enhance *routine* use of a tool box for improving quality of emergency care for acute myocardial infarction (AMI), measures of the related implementation plan realization might include the following:<br>o Proportion of staff reached both by introductory/persuasive mailings and educational presentations from a local champion (and the extent to which the latter included originally intended content)<br>o Observational or self-reported degree of access to and completion of web-based education/skill programs<br>o Observational or assessed degree to which needed organizational systems and leadership support were available or made available in the form of sufficient computers to access the web-based program and in the form of a new organizational policy re: a call system<br>o Perceived value of the various adoption interventions, such as the web-based skill program and local champion.	• *Key aspects* of evaluating an implementation plan include the following:<br>o Operationalization: i.e., the degree to which each of the chosen implementation interventions was actually put into place<br>o Fidelity: i.e., the extent to which there was sufficient conformity in actual implementation relative to the adoption plan<br>▪ *Dose* can relate to operational fidelity and a needed level of intensity. Without such information, it may not be possible to accurately interpret findings or the actual degree of success; e.g., in terms of a Type III error [20].<br>o Core elements; i.e., the extent to which key components of the implementation plan needed for successful replication of the *implementation* strategy were identified and present<br><br>• *Influential factors or needed organizational bases for change* related to the degree of adoption need to be identified and assessed as to whether they were appropriately addressed; e.g., key barriers or enablers that were critical to success, which may have been modified, buffered, enhanced or by-passed:<br>o In reporting on the role of *contextual factors*, designate the following as possibly important to replication:<br>▪ Type of EBP change (e.g., provider level change and/or clinical delivery system level change)<br>▪ Type of clinical setting<br>▪ Organizational resources, systems or values that needed to be present to support uptake of the EBP innovation.	- What dose was delivered for each relevant implementation intervention, including the facilitator role/s?<br>o Was the dose sufficient?<br>o Was the needed dose variable in different situations?<br><br>- To what extent was the use of formative evaluation (FE) ---as an intervention itself---critical to optimization of the change effort and degree of success?<br>o Did emergent refinements to the implementation plan effectively address identified barriers?<br>o Might such barriers have been anticipated?<br>o Do the above suggest refinements to the basic strategy for replication?<br><br>- In terms of the use of a *Facilitator as an intervention*, consider the following:<br>o To what extent was/were the facilitator role/s realized?<br>o What role expectations and activities were most critical for project replication?<br>o Which skills/attributes were most important in what context?<br><br>- How did different stakeholders perceive i) usefulness of the EBP change by the end of the study and ii) usefulness of various implementation interventions for future settings?<br><br>- Per *Theory usefulness*:<br>o Was the stated purpose/s for using the PARIHS framework realized?<br>o How in actuality was the theory used?<br>o Upon critical reflection, how useful was the diagnostic evaluation per PARIHS elements?

		• *Theory usefulness:* From an implementation science perspective, this is an SI indicator that relates to the role of theory in the implementation plan.	o How many and which of the elements/sub-elements were perceived as critical to achievement of various results? Why?<br>o Was a relationship between the framework and "success/progress" clearly evident?<br>o Were there significant gaps in the framework relative to identified barriers and facilitators to "success"?<br>o To what degree, overall, was the revised PARIHS framework user-friendly and helpful in:<br>• Planning the implementation strategy<br>• Conducting evaluations<br>• Identifying barriers and unanticipated elements critical to SI<br>• Enhancing understanding of the black box of the findings, including dynamic relationships and the complexity of implementation?

Related Sub-Elements (Focus of Evaluation)	***Conceptual Definitions***	**Detailed Observations/Tips regarding Sub-elements**	**Sample, Optional Questions to Guide Evaluation [20]**
EBP innovation uptake	*EBP innovation* = An evidence-based clinical intervention and/or delivery system intervention targeted for implementation. Includes provider inclusion of health promotion interventions.<br><br>*Uptake* = Degree to which the desired change in clinical practice is made by the targeted audience/system, congruent with the EBP recommendation.<br>▪ This includes use of targeted patient treatments and/or use of a new approach to delivering targeted care to patients or families<br><br>▪ For example, in the AMI project cited above this might include process measures such as the following:<br>o Proportion of eligible patients ordered and receiving a diagnostic EKG within the guideline specified timeframe<br>o Indication of a new policy re: use of a single call-number to activate cardiac catheterization staff<br>o Awareness of that policy among stakeholders or actual use of that single call-number<br>o Direct observation of other expected physician practices.	• If achievement of innovation results are dependent upon the following, they may also have to be measured:<br>o Increased knowledge or skill and/or a change in attitude, belief or awareness at the individual level<br>o Changes in targeted organizational policies, resources, processes, systems or other entities prerequisite to a change (adherence) in clinician behaviors and/or actualization of a targeted delivery system<br><br>• Key aspects of evaluating the uptake of EBP innovations might include the following:<br>o *Pre-requisite basis for adoption*: i.e., awareness, knowledge, skill or value<br>o *Uptake, adoption or adherence*: i.e., actual use of the EBP on a routine basis<br>o *Fidelity*: i.e., conformity in the *practice* change relative to the core elements of the original innovation<br>o *Sustainability* [24]: i.e., maintenance over time of the innovation<br>o *Diffusion*; i.e., informal spread or uptake/adoption by those not directly targeted within the implementation project<br><br>• Miscellaneous observations:<br>o There are two types of fidelity, applying to more than one indicator of SI; i.e., to uptake and to implementation realization<br>o Process/practice uptake results can be considered intermediary and focus on targeted changes that have to occur first in order to directly create patient outcomes, financial outcomes or other organizational end point outcomes.<br>o It is possible that not only instrumental uptake of innovations is the desired interim outcome, but so too may the following: i.e., cognitive use of evidence or symbolic/strategic use of evidence [25,26]	• To what extent do the targeted clinicians have sufficient, knowledge and skill to perform the EBP?<br><br>• To what extent have the targeted clinicians adopted the EBP?<br><br>• To what extent is the EBP innovation used in routine practice?<br><br>• To what extent is the EBP, as practiced by targeted clinicians, consistent with all of the core elements of the original evidence base?<br><br>• To what extent is the new policy, as tailored by the site, consistent with the core elements of this EBP?<br><br>• To what extent has the adoption persisted, and has the level of adoption been maintained/improved, after ________ (months)?<br><br>• To what extent has the EBP innovation spread to other clinicians or units not targeted?

Related Sub-Elements (Focus of Evaluation)	Conceptual Definitions	Detailed Observations/Tips regarding Sub-elements	Sample, Optional Questions to Guide Summative Evaluation
Patient and organizational outcomes achievement	*Outcomes achievement* = The impact or outcome expected to follow adoption or use of the targeted EBP recommendation. Such outcomes typically include one or more of the following:<br>o *Patient outcomes*: i.e., ultimately desired outcomes expected to result from the EBP innovation<br>o *Financial outcome*: i. e., economically-related implications for the organization<br>o *Staff outcomes*: i.e., end results that reflect issues critical to human resources, and thus the organization<br><br>• For example, in the dissemination of the AMI toolbox, the following measures might be included:<br>o Patient outcomes: 30-day readmission rates and mortality rates for AMI patients; or, organizationally, proportion of AMI patients receiving best practice (i.e., receiving appropriate care for all guideline recommendations)<br>o Financial outcomes: cost per patient with AMI, with and without readmission<br>o Staff outcomes: staff satisfaction with the new care delivery system	• This aspect of measurement is usually referenced as a summative, versus the prior formative evaluation.<br>o Summative evaluation can use and build upon a progress-related outcome; i.e., the same indicator may be used in two different ways (formatively and summatively) at different times [20]<br><br>• End outcomes, versus the interim outcomes of uptake, typically include patient self-management; patient health parameters or quality of life (QOL); and organizational level indicators, such as:<br>o A business case for or against an innovation (budget impact analysis [22])<br>o Especially for a new delivery system, staff burnout or satisfaction.	• To what extent were the desired patient outcomes, expected to result from the EBP innovation, achieved?<br><br>• To what extent was a business case established for the innovation or other cost-related goals achieved?<br><br>• To what extent has staff satisfaction changed with introduction of new role expectations with the EBP delivery system?
